# Bifilm Defects in Ti-Inoculated Chromium White Cast Iron

**DOI:** 10.3390/ma13143124

**Published:** 2020-07-13

**Authors:** Malwina Dojka, Marcin Stawarz

**Affiliations:** Department of Foundry Engineering, Silesian University of Technology, Towarowa 7 str., 44-100 Gliwice, Poland; marcin.stawarz@polsl.pl

**Keywords:** bifilm, chromium cast iron, Ti, inoculation, wear, impact strength

## Abstract

In recent years, white chromium cast iron has gained a well-settled position among wear-resistant materials. In recent times, chromium cast iron samples containing titanium have attracted attention. In cast iron samples, titanium combines with carbon and forms TiC particles, which may be form a crystallization underlay for eutectic M_7_C_3_ carbides and austenite. Accordingly, the inoculation process occurring in the crystallizing alloy should result in the proper, regular distribution of fine eutectic chromium carbides in the austenitic matrix. The presented research was conducted on 20% Cr hypoeutectic white cast iron with the addition of 0.5, 1, and 2% of Ti. Ti inoculation and the presence of TiC allowed for superior wear properties to be obtained. However, the conducted study revealed a significant decrease in the impact strength of examined alloys, especially for the cast iron samples with a high amount of Ti, in which the TiC compounds agglomerated. Titanium compounds accumulate in clusters and their distribution is irregular. Most of the TiC compounds were transported by the crystallization front into the center of the castings, where micropores were formed, meaning they were no longer effective crystallization underlays. In the authors’ opinion, the agglomerate formation is strictly connected with the appearance of bifilm defects in the casting microstructure. The conducted research shows how an incorrect volume of an additive may have negative influences on the properties of the casting. This is a vital issue not only from a technological point of view, but also for economic reasons.

## 1. Introduction

**Figure 1:**
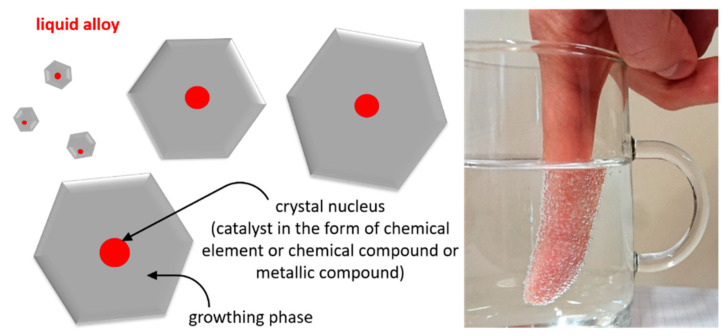
Schematic diagram of the effects of nucleogenic elements on the growth of crystals.

The source literature explains the existing research outlook regarding the modification of chromium cast iron [1,2,3,4,5,6,7,8,9,10,11,12]. A large number of publications describe the effects of nucleogenic elements after their addition to liquid metal, typically in the form of ferroalloys, which stimulate the creation of hard-to-melt compounds as crystallization underlays for primary austenite and chromium carbides (inoculation process), depending on the selected cast iron chemical composition [1,2,3,4,5,6,7,8,9]. Their use increases the number of eutectic colonies in the microstructure, with carbides featuring more favorable morphologies. As was mentioned in previous works [2,3], in order to create an effective underlay the mismatch δ between lattice parameters of the underlay and the phase that one wants to inoculate has to be low. The effectiveness of inoculation decreases when the mismatch δ is higher than 6%. Previous studies have shown that the high melting point of TiC allows its formation before the crystallization of M_7_C_3_. The combination of early crystallization with the low mismatch between lattice parameters of TiC and Cr_7_C_3_ makes titanium carbide a suitable crystallization underlay for eutectic chromium carbides. Research has proved the positive influence of TiC formation on the crystallization and wear resistance of chromium cast iron. According to Bedolla-Jacuinde et al. [1,4,5], the addition of Ti refines the microstructure of chromium cast iron, since the TiC precipitation creates crystallization underlays for primary austenite. In [4,5], the authors state that the addition of an Fe-Ti-RE-Bi mixture changes not only the morphology of austenite, but also the eutectic carbides. The microstructure improvement causes a decrease in the wear rate. Similar effects on the microstructure and wear behavior of chromium white cast iron were achieved by other researchers [6,7,8,9]. Chung et al. [6] proved that the addition of carbide forming elements V, B, and Nb may result in enhanced performance under pin-on-disc testing conditions. Interesting results can be obtained after adding rare earth elements such as Ce and La into the melt [3,4,5,10,11,12]. The lattice parameters of Cr_7_C_3_ carbides are *a* = *b* = 6.99 Å, *c* = 4.036 Å; while the parameters of Ce_2_O_2_S are *a* = *b* = 6.942, *c* = 4.036 Å (*δ* = 0.69) in a hexagonal system [3,10]. The low mismatch δ between lattice parameters of Ce_2_O_2_S and Cr_7_C_3_ makes the Ce_2_O_2_S compounds suitable crystallization underlays for Cr_7_C_3_ eutectic chromium carbides. Studies in this area show that the addition of rare earth elements improves the morphology of the carbide phase and make carbides more rounded and refined [3,10,11,12]. The microstructure refinement after the addition of Ce and La, resulting in better wear resistance [3,4,5]. However, a higher amount of Ce lowers the impact strength [10,11]. The inoculation process most frequently improves the mechanical properties and performance of chromium cast iron, however introducing additional alloy ingredients in the form of hard carbides such as TiC, NbC, and VC, as well rare earth elements (REE), stimulates the abrasive wear resistance, which can make the alloy more brittle [5,6,7,10,11]. Figure 1 presents a diagram of a nucleogenic modifier operation and a photo that perfectly clarifies the concept of creating crystallization nuclei on an underlay.

The compounds and elements introduced into the melt affect the crystal nucleation in alloys via modifiers, as in the case of the finger immersed in fizzy water shown in the photo, where the “nuclei” on its surface appear in the form of carbon dioxide particles. The modification process for chromium cast iron is interpreted in different ways. The literature states that only a small amount of modifier should be used to avoid considerable changes in the chemical composition and adding extra microstructure components. Some publications recommend adding elements at the quantity of up to 5% by weight to modify the structure. Here, only the nucleogenic modification is considered necessary to create crystallization underlays. Jura [13] defined the modification identifies of modifiers with alloy additives, stating that “the modification process consists of introducing certain alloy (modifying) additives into the melt. The additives, referred to as modifiers, stimulate a change in the primary structure”. The modification process is defined mainly by the quantity of modifiers added, their type, the method of introduction, and particularly the final effect. The reason for using modifiers is that they can change the structure and improve the performance of casting products. These results satisfy casting manufacturers.

Studies on grey cast iron [14,15,16] have shown that increasing the amount of titanium in an alloy may increase the formation of primary austenite dendrites. The addition of titanium into the melt increases liquidus temperature by forming the crystallization underlays for primary austenite. The addition of a small amount of titanium creates fine interdendritic graphite. The latest research by Alonso et al. [17] indicated that Ti carbonitrides that precipitate on Mg-Ca sulfides and inhibit their growth could be the perfect crystallization underlays for graphite nucleation. Opinions differ regarding the mechanical properties of grey cast iron. Larranaga et al. [16] mentioned that the addition of 0.3% Ti in grey cast iron may improve the UTS (ultimate tensile strength) of the alloy, while others point out that the content of titanium should not exceed 0.075% because higher amounts can lead to decreased strength. Another study [16] confirmed that the addition of Ti up to 0.4% with a low content of S in grey cast iron significantly increased the tensile strength. The addition of titanium via microalloying has an important role in the production of high-strength steels. Ti in steels influences the grain refinement strengthening and precipitation strengthening [18]. As a result, titanium microadditions increase the yield strength and impact toughness of steel plates and line pipes.

The basic objective of this paper was to analyze the nucleogenic effect of Ti addition on the microstructure shape, including the stereological parameters of carbides in chromium cast iron and the influences of additives on the abrasive wear resistance, hardness. and impact strength of experimental alloys.

## 2. Materials and Methods

### 2.1. Casting Material

**Figure 2:**
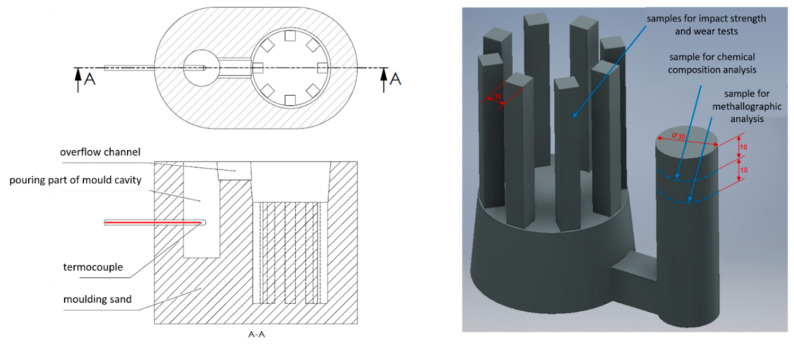
Scheme of the mold cross-section (ATD-P tester) and a 3D casting model [19].

The melting batches were performed in a medium-frequency laboratory induction furnace (PI25, ELKON Sp. z o.o., Rybnik, Poland) with a capacity of 25 kg. The experimental melts used only a small amount of charge material (about 12 kg). To maintain a uniform pouring temperature and avoid undercooling (solidification) of the alloy in the ladle, the temperature was also measured in the mold used to make samples for impact strength and abrasive wear resistance tests. For this purpose, ATD-P and ATD-Pi molds were designed [19]. Figure 2 presents the scheme of the mold and a 3D casting model with marked sampling locations for individual tests. Samples were taken for metallographic, impact strength, abrasive wear resistance, and alloy hardness tests.

During the melting process, a carburizer was added to the charge material of initial cast iron (W0) to obtain a near eutectic chromium cast iron composition. The melt was deoxidized by transferring the heated ladle with Al (0.1% by weight) placed on the bottom, then FeTi was placed at the furnace bottom, the ladle content was repoured into the furnace, and after reaching an adequate temperature (1550 °C) the melt was poured into the ladle and the molds were filled. This made it possible to avoid the influence of a large number of additives on the metal’s surface when transferring it into the ladle, which would result in a considerable modifier depletion in the liquid. Table 1 presents a list of the chemical compositions of experimental cast iron samples obtained from the spectrometric analysis using a LECO GDS500A spectrometer (Model No607-500, LecoCorporation, 3000LakeviewAve, St. Joseph, MI, USA).

### 2.2. Metallographic Examination

The structure of the experimental chromium cast iron samples was tested in a few stages. Preliminary metallographic tests were conducted by using the light microscopy at the same time as taking microstructure photos for the purpose of carbide-phase stereological analysis. Then, the measurements of the chromium carbide share were performed. Testing of the average surface area, length, and width of chromium carbide in experimental cast iron samples was conducted to confirm the effectiveness of the additions on the fine microstructure creation. For certain alloys, their microstructures were analyzed using scanning electron microscopy (SEM). Sample fracture surfaces of impact-tested specimens were analyzed at the macroscale, while certain fractures were tested using scanning electron microscopy (SEM). Samples measuring 30 mm in diameter were taken from test castings (see Figure 2) and specimens were prepared for metallographic tests by wet polishing using sandpapers, buffing, and etching in ferric chloride solution for about 15 s. The microstructure was analyzed using a Nikon light microscope (Eclipse LV150N, Nikon Metrology Europe NV, Geldenaaksebaan 329, 3001 Leuven, Belgium) combined with a camera. The stereological analysis was conducted using NIS Elements software (NIS-Elements Advanced Research, Nikon Instruments Inc., 1300 Walt Whitman Road, Melville, NY, USA). Metallographic tests using the Phenom ProX (Phenome-World Eindhoven, North-Brabant, Netherlands) scanning electron microscope (SEM) covered unetched metallographic samples.

### 2.3. Hardness and Impact Strength Tests

The Charpy impact test was carried out by using a SUNPOC Impact Tester (JB-300B Pendulum Impact Testing Machine, GUIZHOU SUNPOC TECH INDUSTRY CO., LTD., Meidi Fortune Center, Changling North Road, Guanshanhu District, Guiyang, China). In each case, three chromium cast iron samples without a notch were broken. For the sample tests, fractures that showed visible flaws were rejected and the test was repeated. Rockwell hardness tests were performed on the samples, whereby at least three measurements per sample were carried out, then the average values were calculated.

### 2.4. Abrasive Wear Resistance Tests

The process of modifying chromium cast iron is most frequently performed to provide a structure that maintains or improves the resistance to abrasion. To determine the influence of additives on the abrasive wear resistance, experimental tests of alloys were performed using two gravimetric methods in different sample motions (reciprocation and rotation) using the pin-on-disc method. Tests were conducted on prototype devices designed in the Department of Foundry Engineering in Gliwice [20,21].

The abrasion process by pin-on-disc method took place under the following parameters: dry abrasion; the abrasive disk was P80 sandpaper, aluminum oxide; abrasive disk speed was 155 rpm, while the sample holder speed was 400 rpm; the loading per single sample was 220 G. Before starting the test, each sample was pre-abraded to provide a full-face contact area with the abrasive material. After pre-abrading, the initial weight of each samples was determined. To start the test, a sample was installed in the holder on a rotational head, while the abrasive disk was replaced with a new one before starting a new test series. The test consisted of 60 min test series performed in 10 min test cycles. After each cycle, the sample was cleaned and then weighed to check for any loss in weight during abrasion.

During the tests using the other method, the sample was placed in a holder on the arm driven by a 3-phase electric motor moving in a reciprocating motion. Below the tested material, carborundum abrasive sandpaper was attached (SiC) with a grain size of 50, which was used as the counter sample material. The sample face was loaded with a preset load of 10 N. As a result of friction, the samples during the motion were subject to abrasive wear. 

After previous pre-abrading of all the samples to obtain a full-contact interface with the abrasive material, the test started. The abrasive wear test started by weighing all samples on a laboratory scale. A weight was attached (a 1 kg disk) and the machine was preset to 1000 cycles of motion. After 1000 cycles the counter sample was replaced, while the whole measurement included 5000 motion cycles, which provided the total sample travel distance of 1000 m in a reciprocating motion. Then, the sample was weighed and the loss in weight was calculated. The total abrasive wear test was repeated three times and the average weight loss of the samples was calculated based on this.

## 3. Results and Discussion

### 3.1. Metallographic Examinations Results

Metallographic tests allowed us to partly specify the effects of additives and the changes that occur in the growth of crystals after their application.

#### 3.1.1. Light Microscopy Analysis

**Figure 3:**
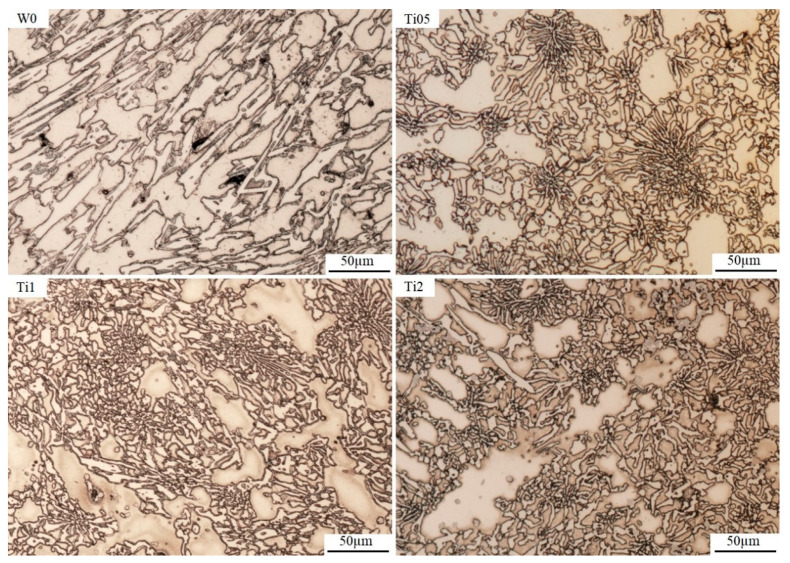
The microstructures of experimental chromium cast iron samples, as viewed under a light microscope.

Figure 3 presents the micrographs of all samples. The photos of the microstructure revealed certain changes occurring during crystallization after use of the additives. For all samples with titanium, a refinement of the microstructure can be seen. The majority of the long, sharp-ended carbides observed in the sample without additives were replaced by the γ + M_7_C_3_ eutectic, consisting of finer chromium carbides. The analysis of the presented micrographs also shows a certain difference in the share of the carbide phase. Unfortunately, a reduction in the volumetric share of carbides is particularly noticeable in samples with Ti added, while their quantity is reduced as the element concentration increases. This results from the formation of titanium carbides (TiC) containing large amounts of carbon in the structure of the chromium cast iron. Based on the conducted metallographic analysis, we can say with high probability that the phases with Ti constitute crystallization underlays for austenite and eutectic M_7_C_3_ carbides. They occur both inside the chromium carbides and the austenitic matrix. Any reduction in the carbide phase content also results in the appearance of austenite dendrite arms visible on the photos, which are particularly noticeable on samples with 2% addition of Ti. The presented metallographic tests provide relevant information on the use of titanium as an additive for chromium cast iron. If the chromium cast iron eutectics contain titanium carbides, this means that titanium absorbs a large amount of C from the liquid needed to create compounds with carbon. To maintain the eutectic composition of the alloy and simultaneously maximize the fragmented structure, the carbon level in the alloy can be replenished by carburization. The analysis shows a considerable increase in the amount of titanium compounds with carbon as the titanium concentration grows. In samples with 0.5% Ti added, the compounds are almost unnoticeable. It is worth mentioning that the visible refinement of the microstructure was obtained for the Ti05 sample, in which the addition of titanium was significantly lower than for the other samples. Reducing the quantity of carbon in the liquid also decreases the volumetric share of chromium carbides in the microstructure and results in the appearance of primary austenite dendrites.

#### 3.1.2. Stereological Analysis of the Carbide Phase

**Figure 4:**
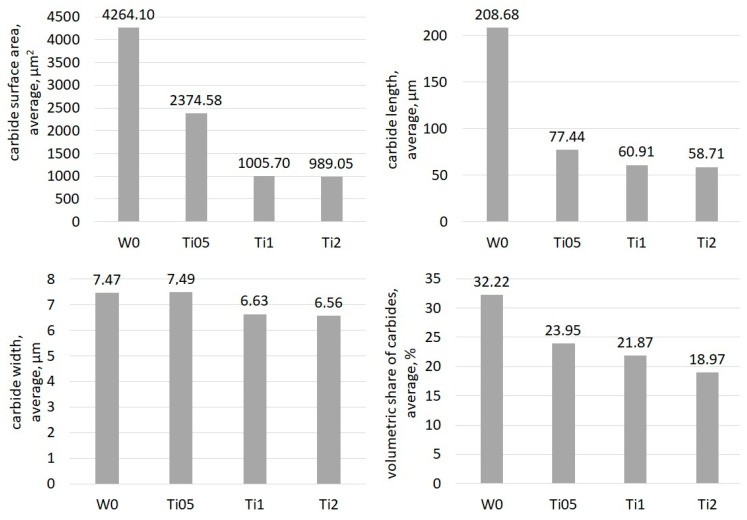
Average surface area, length, width and share of carbides in experimental samples.

The results of selected stereological parameter measurements of eutectic carbides and carbide share analysis are presented in the form of graphs in Figure 4. The presented parameters decrease as the amount of titanium increases, but considering the values of average carbide length, it can be noticed that the largest changes occur for the alloy with 0.5% Ti added (Ti05). The average length of carbide in the Ti05 sample is almost 3 times smaller than the same parameter for the sample without Ti (W0). On the other hand, the difference between Ti05 and Ti1, where the addition amount was 2 times bigger, is only 26%. Moreover, the average surface area of the carbide for the Ti2 sample is only about 2% smaller than the parameter measured for Ti1. A similar difference occurs for the average length value for those samples, which only equals about 3%. Comparison of the alloy containing 1% Ti to the alloy containing 2% Ti shows that in spite of the double modifier level, the sizes of carbides do not noticeably change. The differences in the average widths of carbides in all alloys range do not exceed 1µm. Analyzing the average volumetric share of the carbide phase in the investigated areas allows us to conclude that the use of modifying additives under analysis contributes to the decrease in the carbide phase share in chromium cast iron.

#### 3.1.3. SEM Analysis

**Figure 5:**
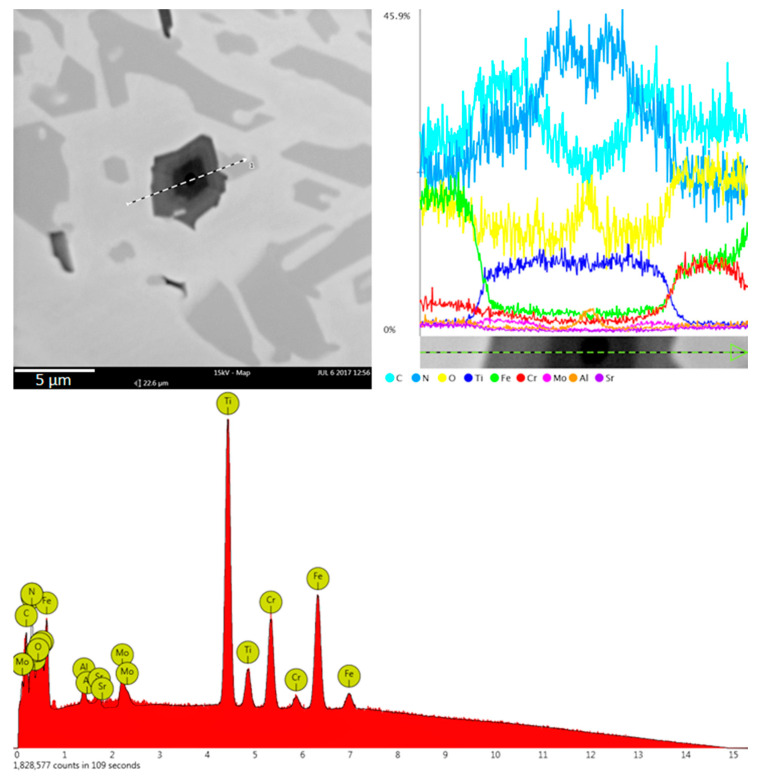
Linear scan of the phase with Ti and other elements in the sample with 1% Ti addition, as viewed with EDS SEM.

**Figure 6:**
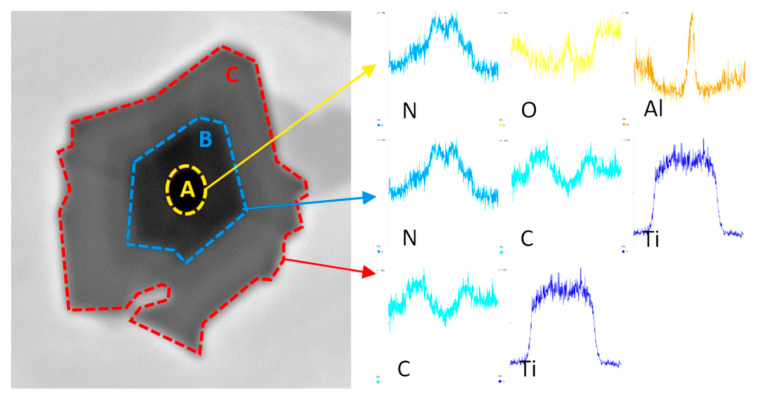
Zones of elements in a complex phase with titanium in the sample containing 1% Ti.

The microstructure of all experimental chromium cast iron samples abounds with γ + M_7_C_3_ eutectics, but also contains the precipitates of primary austenite or hypereutectic chromium carbides. Chromium cast iron samples with titanium added contain phases defined as titanium carbides, which are also visible in microstructure photos under light microscopy. Additionally, phases with aluminum occur in both the alloy without modifying additives and in cast iron samples containing titanium. However, in chromium cast iron with titanium added, the inclusions appear inside the Ti compounds, creating a complex compound of titanium, carbon, nitrogen, and aluminum. An example of analysis of a complex Ti compound with other elements is presented in Figure 5. Figure 6 presents the compounds under analysis divided into 3 zones that are visible under a microscope and linked with the levels of the elements. Analysis of the figures allows us to notice that the titanium carbide defined in the preliminary studies crystallizes on an underlay and is probably the compound containing the titanium, carbon, and nitrogen, which is indicated by elements found in zones B and C (see Figure 6). The underlay for the carbide–nitride under analysis could be aluminum oxide or nitride, as shown in the elemental analysis in zone A. In zone C, there may also be a small amount of molybdenum, as shown in the linear analysis (see Figure 5).

Nitrogen in casts most frequently occurs in the gas form creating bubbles and porosities. The literature [22] states that both aluminum and titanium bind nitrogen, which most frequently leads to restriction of its impact on the crystallization of structure by degassing. However, nitrides that remain after refinement in grey cast iron are considered to act as substrates for graphite nucleation. A similar situation can occur for compounds created in the chromium cast iron under analysis as compared to the nucleating chromium carbides.

**Figure 7:**
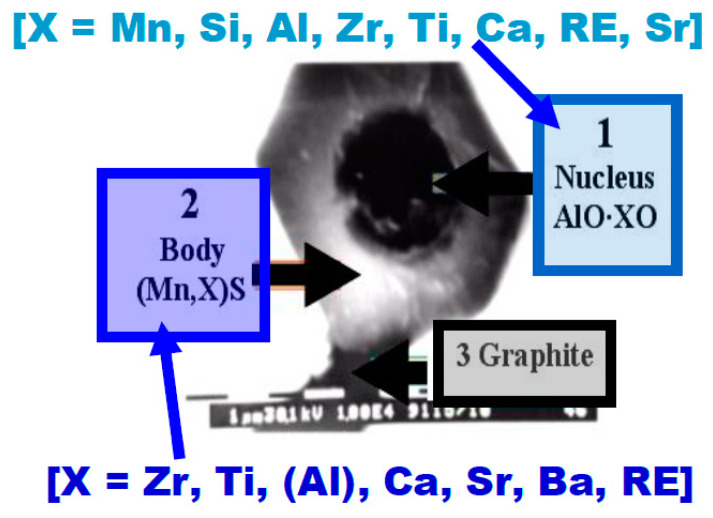
Three-stage nucleation model of flake graphite [23].

The occurrence of nucleation substrates in the form of oxides, nitrides, or sulfides is perfectly described in the studies conducted by Riposan, who analyzed the crystallization of graphite inclusions in grey cast iron [23,24]. Observing the analysis performed by Riposan (see Figure 7) allows us to find similarities to the investigations performed herein.

**Figure 8:**
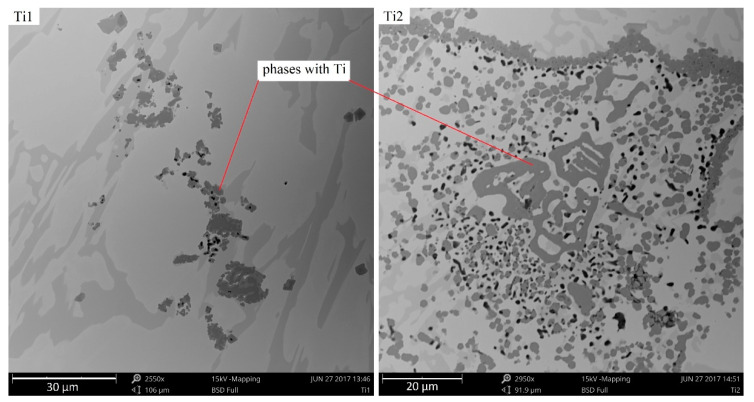
Ti-containing phases in the microstructures of samples with 1% and 2% addition of Ti, as viewed under SEM.

The hard phases of Ti with other elements are not evenly distributed within the structure, especially for alloys with a large amount of Ti added (1%, 2%), creating agglomerations of fine phases adjacent to γ + M_7_C_3_ eutectics. Figure 8 presents distribution examples for complex Ti-containing compounds in the microstructures of the chromium cast iron samples under analysis. The phases clearly vary in size. One alloy contains both smaller and larger precipitations, with cross-section lengths ranging from 2 to 8 μm. Most frequently, the titanium carbides create large agglomerations of different shapes.

**Figure 9:**
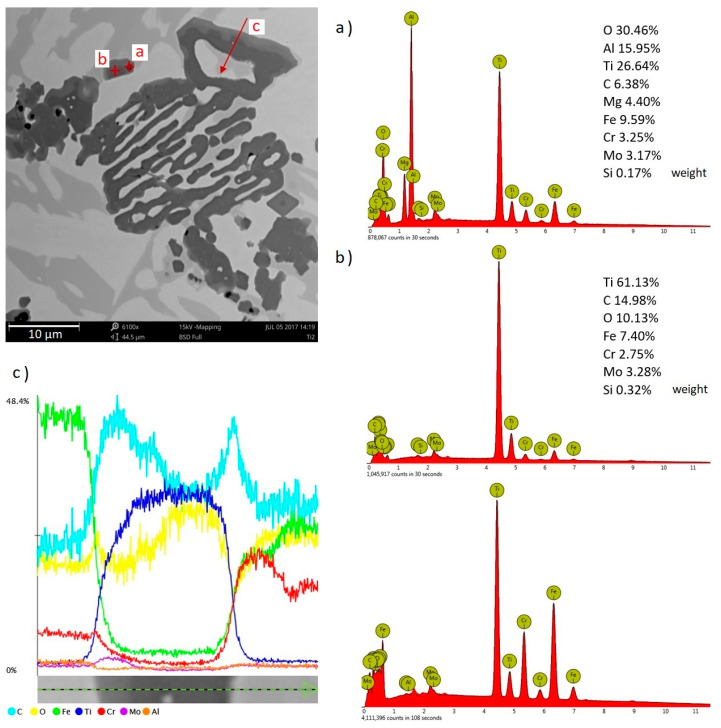
Point (**a**,**b**) and linear (**c**) EDS analyses in the areas of agglomerates with Ti-containing phases in a sample containing 2% Ti, as viewed under SEM.

Figure 9 presents linear and point EDS analyses performed in the areas of phases with Ti agglomerations. The small darkest areas observed in the photo of the Ti2 sample are aluminum compounds adjacent to the complex agglomerations of titanium carbides, as confirmed by analyzing item a) in Figure 9, which can be identified by the analysis of item b) and the linear EDS analysis c).

**Figure 10:**
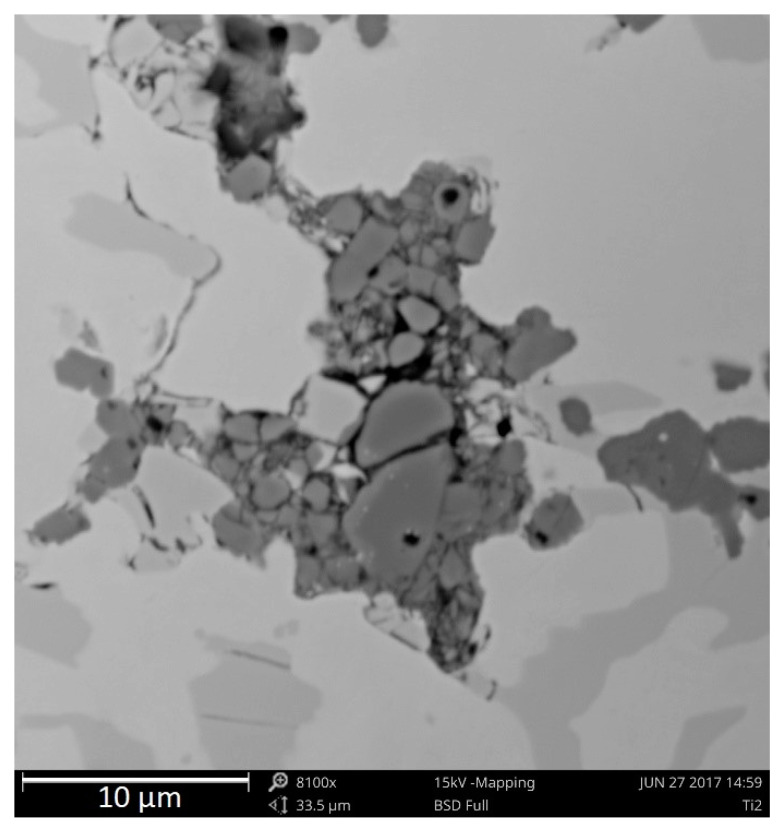
Phases with Ti closed in a hollow space, as viewed under SEM.

The agglomeration of small phases with titanium can be linked to their crystallization on the underlay in the form of precipitations with aluminum. However, while preparing the paper, another hypothesis of phases with titanium agglomeration was conceived, which was linked to the method of pouring and filling a tester mold cavity and the melting course conditions. Figure 10 presents a SEM photo of titanium carbide agglomerations closed in a hollow space (not surrounded by a matrix) observed in a metallographic Ti2 specimen.

Near the agglomerate of phases with Ti (see Figure 10), a dark line can be seen that looks like a crack. However, if this phenomenon is linked to the turbulent filling of the mold and the inclusions developing in the liquid metal, one can assume that the carbides under observation could have been tangled in the non-metallic inclusion (in the form of a bifilm, constituting a trap for the carbides) while filling the mold or during casting crystallization, and the supposed crack visible on the left may be a part of this inclusion. Closing titanium carbides in the bifilm can be caused by turbulence and the dynamics of the pouring process, convection currents, or flotation. The density of titanium carbides is about 4.91 g/cm^3^ and is much lower than the density of liquid chromium cast iron (approximately 6.6–7.1 g/cm^3^). The difference in density makes titanium carbides susceptible to flotation, as in the case of graphite in hypereutectic grey cast iron, which also shows this property. In both cases, these phases are formed at the early stage of crystallization.

**Figure 11:**
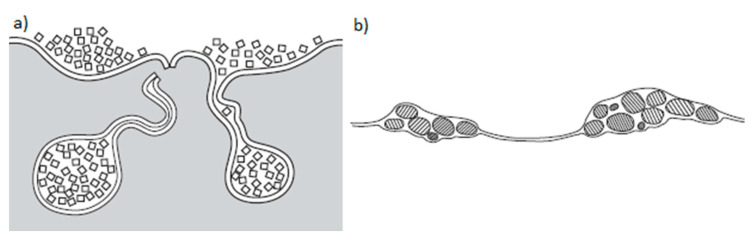
Mechanism of agglomerate creation in particles closed in the inclusions (**a**) and an example of inclusion in the alloy (**b**) according to Campbell [25].

**Figure 12:**
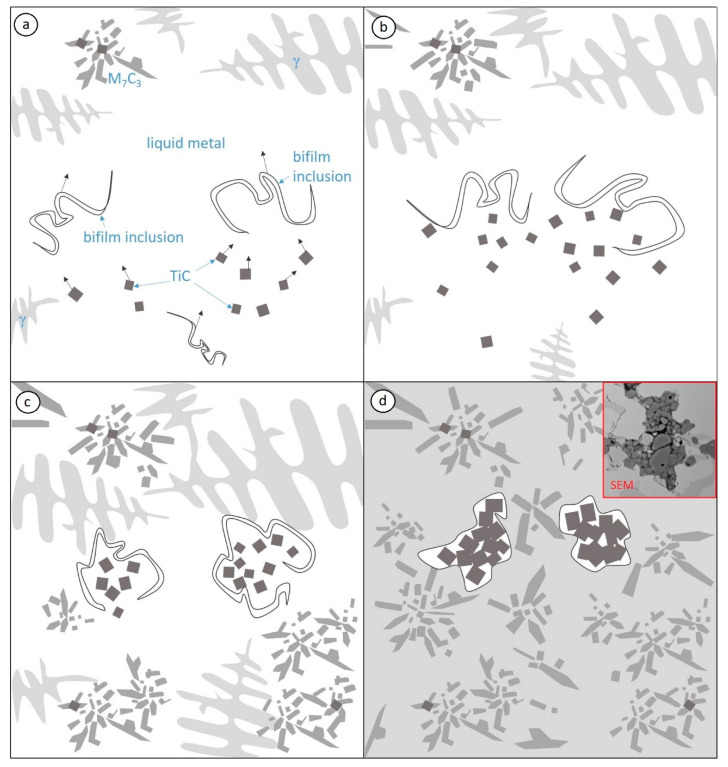
Schematic diagram presents four steps (**a**–**d**) of formation of agglomerates with Ti-containing phases according to the closure hypothesis in bifilm inclusions.

The discussed phenomena linked to the turbulent flow of liquid were described in detail by Professor John Campbell [25]. Figure 11 presents his schematic diagram of closing ceramic particles in inclusions during the turbulent pouring and filling of the mold cavity. A similar phenomenon can occur for TiC agglomerates; therefore, based on Professor Campbell’s theory, a hypothesis for the creation of titanium carbide (TiC) agglomerates under observation was developed. The schematic diagram of TiC agglomerates as supposed by the hypothesis of the TiC closure mechanism in inclusions, as presented by the authors of the paper, is presented in Figure 12.

The hypothesis for the creation of agglomerates and phases with Ti by closure of the inner bifilm inclusions considers the fact that the molten metal involved in filling the mold already contains titanium carbides (TiC) and inclusions of a lower density than the liquid. This difference in density, the convection currents, and the pouring dynamics cause titanium carbides (TiC) to move in the molten alloy (see Figure 12a). The floating light carbides encounter bifilm inclusions in the molten metal (see Figure 12b). Phases with Ti are closed within the inclusions and are moved up or pushed away by the crystallization front, until they encounter an emerging crystal (see Figure 12c). This mechanism may be adopted by TiC agglomerates observed in the microstructure (see Figure 12d). Ti-containing phases occur near eutectic chromium carbides and in the austenitic matrix. Based on the microstructure tests performed, one cannot come to an unambiguous conclusion concerning the crystallization-inducing nature of those Ti-containing phases. At the same time, these phases constitute an underlay for austenite nuclei and γ + M_7_C_3_ carbide eutectics.

#### 3.1.4. Fracture Analysis

**Figure 13:**
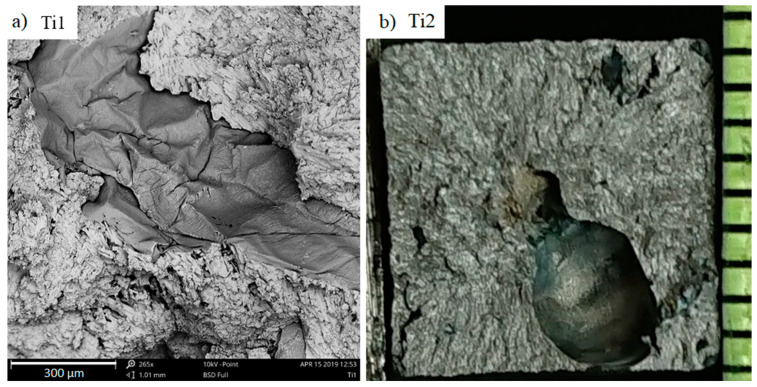
Undulating inclusion film in a chromium cast iron sample with 1% titanium, as viewed under SEM (**a**); gas bubbles in the sample with 2% titanium (**b**).

The fracture surfaces of the investigated samples have a brittle character, which is typical for high-chromium cast iron. The macroscopic metallographic analysis allows us to notice numerous oxide inclusions, especially in samples with added titanium. Figure 13a presents magnifications of undulating oxide films that appear in samples. In certain samples, large gas bubbles can also be seen in the fractures (Figure 13b).

**Figure 14:**
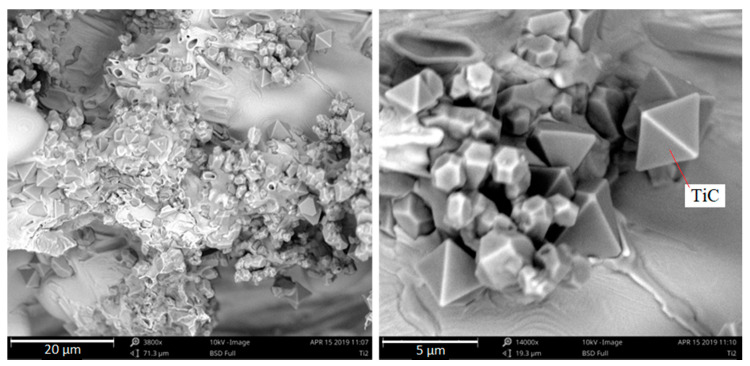
Phase with titanium (TiC) in austenite dendrites and TiC agglomerates in shrinkage cavities in the fractures of samples with 2% Ti, as viewed under SEM.

The vertical axis of each sample shows a shrinkage cavity that is created in the crystallization process. The surface area is especially highly developed for the samples containing high titanium concentrations. The dendrites of austenite observed in the contraction cavity show numerous titanium carbide (TiC) precipitations (Figure 14).

**Figure 15:**
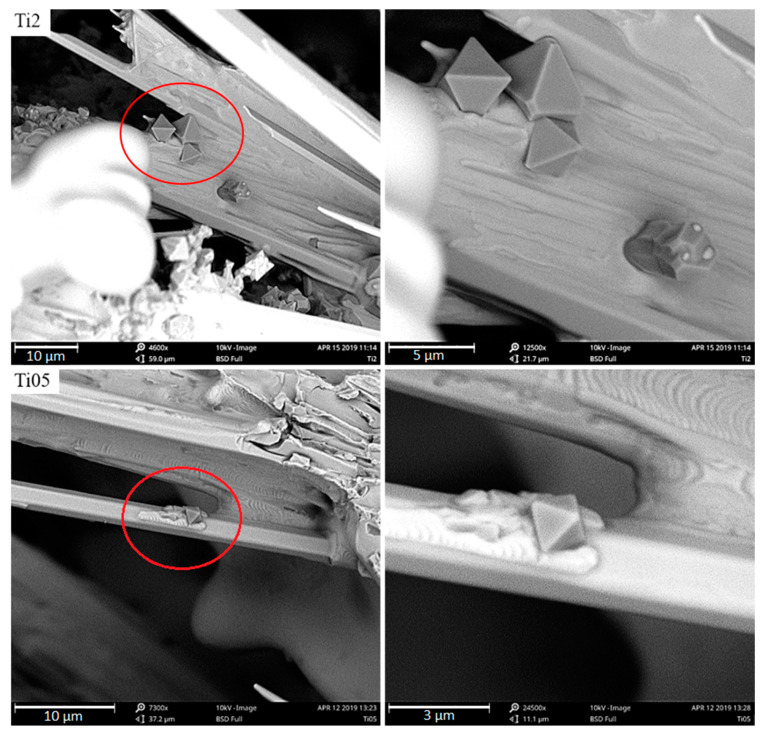
Titanium carbides in the eutectic grains of the Ti2 sample and in the primary chromium carbide in the Ti05 sample, as viewed under SEM.

Observing Ti-containing phases this way makes it easier to verify their shape. In the samples under analysis, they adopt an octahedral shape, i.e., a regular octahedron. At higher titanium concentrations, the compounds accumulate more intensely, creating huge agglomerations that appear to have been pushed into the crystallization front, together with inclusions towards the casting center. When the supply of liquid metal feeding the crystallizing casting ends, they remain in the axial pores of the sample in austenite dendrites. The majority of titanium carbides occur in austenite dendrites, as shown in SEM micrographs. This analysis suggests that the TiC in the examined chromium cast iron is mainly a crystallization underlay for austenite. Coming back to the analysis of the chemical composition and the crystallization analysis presented in other papers [2,3], this theory can be accepted because the results of the crystallization study show that the obtained alloys are hypoeutectic or nearly eutectic chromium cast iron samples, where austenite crystallizes as the first phase. Nevertheless, the nucleogenic action of TiC on M_7_C_3_ carbides is not excluded due to the similarity of the crystallographic grid parameters mentioned in the introduction and the results of studies provided by domestic and foreign scientists. The tests of metallographic samples also indicate the presence of Ti-containing phases together with γ + M_7_C_3_ eutectics and inside the eutectic chromium carbides. On the surface of the fracture, no titanium carbides were noticed. Figure 15 presents Ti-containing compounds in eutectic grains and primary chromium carbides.

**Figure 16:**
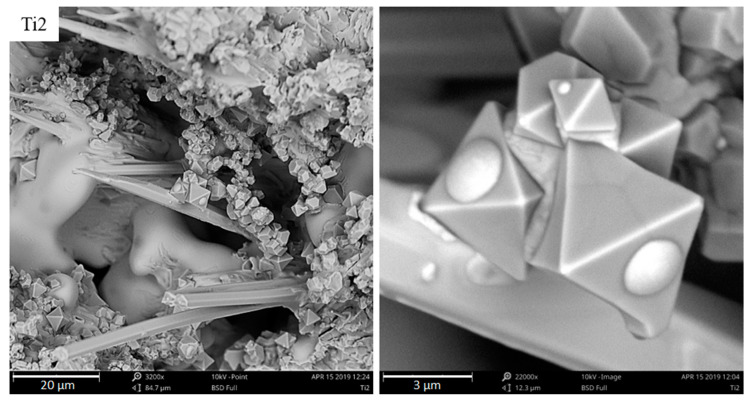
The beginning of dendrite growth on titanium carbide in the axial casting pores, which is pushed into inclusions by a crystallization front, as viewed under SEM.

Figure 16 shows a large magnification of the accumulated Ti-containing compounds in axial pores of a sample with 2% titanium added, where the austenite begins crystallizing. However, its growth is stopped due to the lack of liquid metal feeding the shrinkage.

By summing up the abovementioned study results, we should question the addition of high quantities of titanium for modification purposes. Analysis of the provided SEM microphotos allows us to conclude that titanium’s nucleating potential is reduced after exceeding 1% by weight of Ti addition. Titanium carbides introduced into the mold cavity constitute effective crystallization substrates to a certain degree, while the remaining Ti carbides show no positive influence on the properties of chromium cast iron because they are pushed away by the crystallization front.

### 3.2. Hardness and Impact Strength Test Results

**Figure 17:**
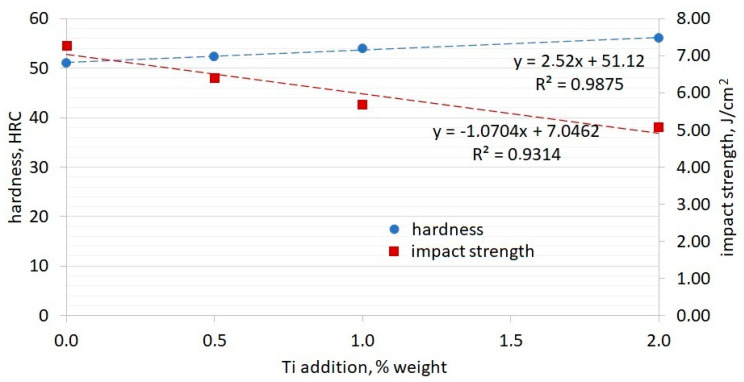
Hardness and impact strength test results for samples with addition of Ti.

The Rockwell hardness test results are presented in Table 2. The average HRC (Rockwell hardness) values and results of impact strength tests are presented in the graph in Figure 17.

By analyzing the values presented in Table 2 and the graphs, one can notice that an increase in the quantity of titanium in the alloy causes an increase of HRC for the chromium cast iron under analysis. The table shows high deviations between individual measurements, which could indicate a local alloy hardening caused by addition of titanium-containing phases in the microstructure. If the indenter hits the area containing TiC agglomerates, the HRC values are much higher. As per the presented graph, the addition of titanium decreases the impact strength of chromium cast iron under analysis. The lowest impact strength is for experimental cast iron containing 2% Ti, which is 30% lower than the impact strength of initial cast iron (W0), without modifying additives. The value of the R^2^ determination coefficient value indicates a link between the impact strength value and the quantity of the modifying element added in those four cases. The linear dependency also appears for the hardness in terms of the Ti additive quantity. The addition of 1% or 2% titanium had a meaningful impact on the bifilm formation, which was visible in the fractures of samples. Considerable changes in the eutectics of alloys and the creation of agglomerates of Ti compounds could significantly affect test results.

### 3.3. Abrasive Wear Resistance Analysis

**Figure 18:**
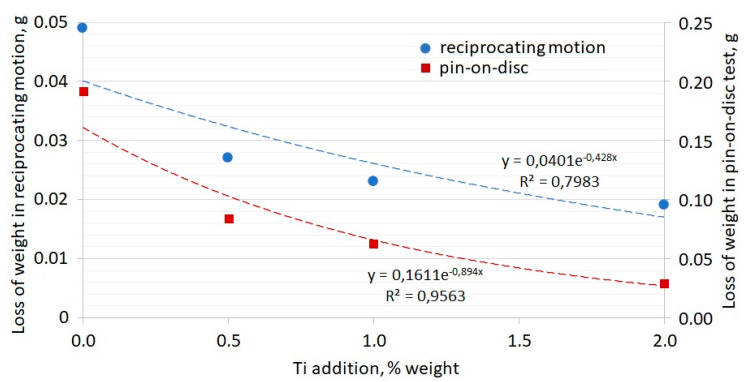
Losses in sample weight for the experimental chromium cast iron samples during an abrasive wear test in reciprocating motion and pin-on-disc tests.

Figure 18 shows a graph presenting the losses of weight for chromium cast iron samples, depending on the quantity of the modifying addition for the two test methods. Analysis of the results presented in the graph allows us to conclude that the additions reduce the abrasive wear of the chromium cast iron under analysis. However, the non-proportional impact of Ti additives on improving the abrasive wear resistance of the cast iron under analysis is noticeable. It is worth noting that the differences in weight loss between samples with 0.5% of Ti, 1% of Ti, and 2% of Ti are reduced compared to the differences of weight loss between samples without Ti (W0) and with 0.5% of Ti. This is why doubling the Ti addition amount from 0.5 to 1% and from 1 to 2% does not provide constant effects in reducing abrasive wear. There is no clear linear dependency. The values of weight loss change exponentially. This is especially visible for the results from the pin-on-disc test. Doubling the quantity of the Ti modifier does not result in doubling the weight loss reduction.

Here, we should return to the results of metallographic tests showing an uneven distribution of compounds created by titanium in the alloys under analysis. If the sample was cut in the area containing a large share of hard TiC or whole agglomerates, the abrasive wear resistance in such a place would be higher, whereas the loss in weight would decrease. The agglomerates of titanium carbide begin to appear as the quantity of Ti addition increases, which may explain the differences in the abrasion test results. These titanium compounds play a bigger role in the abrasive wear of the cast iron samples under analysis than the change in carbide morphology after modification with the addition of 0.5% Ti. Thus, if we do not obtain even distribution of the phases with Ti in the casting microstructure after higher addition of Ti, the increase of the wear resistance will not be proportional. Moreover, the same disproportional increase was observed in the stereological analysis of eutectic carbides, which is proof that the wear resistance of chromium cast iron samples with 1 and 2% Ti is mostly increased by TiC formation and not by a change in the carbide morphology. Nevertheless, if manufacturers decide to use high inoculant levels to create crystallization underlays and hard phases to increase the wear resistance, they should consider quality casting technology. The design of a proper gating system could help to eliminate bifilm defects [26,27,28,29,30,31,32,33,34], which generate TiC agglomeration. This may improve the TiC distribution, providing more even wear properties in the entire casting. The decreased impact strength could also be avoided via low-inclusion casting.

## 4. Conclusions

The modification of high-chromium cast iron with titanium affects the casting crystallization, while the analysis of quantitative and qualitative microstructure tests showed that Ti may form the crystallization underlays for austenite and chromium carbides. The Ti inoculation process allows the fine microstructure of the chromium cast iron to be obtained. Titanium carbides increase the hardness and the abrasive wear resistance of chromium cast iron; however, using higher Ti levels (over 0.5%) to stimulate its creation unfortunately does not provide the expected effects, as the distribution of the titanium compounds is impaired. They start accumulating into agglomerates in bifilm inclusions and migrate at the crystallization front, while a large amount of the Ti does not create effective crystallization underlays and does not improve the abrasive wear resistance. Increasing the titanium addition over 0.5% results in creation of the agglomerates of Ti compounds (TiC), which affect the impact strength value and abrasive wear resistance of high-chromium cast iron, thus stopping the inoculation and microstructure refinement effects. The smallest M_7_C_3_ carbides occur for samples with high Ti additives (1 and 2%). However, the differences in stereological parameters related to the size of carbides between the alloys with 1% Ti and the cast iron with 2% of Ti are insignificant, despite the amount of additive being doubled, which may be due to the reduction in the effectiveness of crystallization underlays during agglomeration of TiC in the casting contraction cavity. Bifilm formation as a reason for TiC agglomeration causes the uncontrolled local increase of wear resistance, while the impact strength of the alloy decreases. Admittedly, in the case of the reciprocating motion test, the losses of weight decreased by 60% and 85% for the pin-on-disc test as compared to the cast iron sample without Ti. However, the abrasive wear tests showed that the difference in weight loss after doubling the amount of titanium from 1% to 2% was insignificant. This is significant for the foundry industry for economic reasons. There is no justification for using such a high level of inoculant if it is no longer effective. This should be considered by manufacturers.

## Figures and Tables

**Table 1:** Chemical compositions of experimental samples by weight % (spectrometric analysis).

	C	Cr	Ti	Mn	Si	Ni	Mo	Al	V	Zr	S	P	Nb	Cu	Fe
**W1**	2.85	20.4	0.01	0.39	0.66	1.48	0.57	0.22	0.13	0.24	0.02	0.05	0.07	0.03	bal*
**Ti05**	2.96	20.5	0.17	0.42	0.85	1.42	0.55	0.18	0.13	0.28	0.02	0.05	0.09	0.03	bal*
**Ti1**	3.12	19	0.46	0.39	0.79	1.49	0.59	0.12	0.15	0.29	0.03	0.05	0.11	0.03	bal*
**Ti2**	3.09	19.6	1.08	0.35	0.82	1.46	0.59	0.16	0.17	0.28	0.03	0.05	0.14	0.03	bal*

*bal = balance.

**Table 2:** Rockwell hardness test results.

Sample	HRC (Rockwell Hardness) Values			HRC Average
**W0**	49	52	52	51
**Ti05**	50	54	53	52
**Ti1**	55	53	54	54
**Ti2**	61	53	54	56
